# Prevalence of Dropout and Influencing Factors in Digital Psychosocial Intervention Trials for Adult Illicit Substance Users: Systematic Review and Meta-Analysis

**DOI:** 10.2196/77853

**Published:** 2025-10-10

**Authors:** Jiayi Li, Xinyi Liu, Xiayu Du, Tingni Mi, Zhihong Ren

**Affiliations:** 1 School of Psychology, Central China Normal University, The 8th floor, Nanhu Complex Building, No.152 Luoyu Road, Wuhan, 430079, China; 2 Key Laboratory of Adolescent CyberPsychology and Behavior (CCNU), Ministry of Education, Wuhan, China; 3 Key Laboratory of Human Development and Mental Health of Hubei Province, Wuhan, China; 4 School of Psychology, Liaoning Normal University, Dalian, China

**Keywords:** digital psychosocial intervention, dropout rate, illicit drug use, meta-analysis, systematic review, influencing factor

## Abstract

**Background:**

Illicit drug use has become a significant global public health issue, and digital interventions offer new approaches to address this challenge. However, there is a gap in existing research on the dropout rate of adult illicit drug users receiving digital psychosocial interventions.

**Objective:**

This study aims to evaluate the dropout rate of adult illicit drug use following digital psychosocial interventions during treatment and the longest follow-up, as well as its predictive factors.

**Methods:**

Following the PRISMA (Preferred Reporting Items for Systematic Reviews and Meta-Analyses) guidelines, studies published up to August 27, 2025, were searched in the Web of Science, PubMed, PsycINFO, Embase, and Cochrane Controlled Trials Register. Randomized controlled trials of digital psychosocial interventions for adult illicit drug users that reported dropout rates were included. Two researchers independently screened studies, extracted data, and assessed bias risk using the Cochrane risk of bias tool (ROB 2.0). A random-effects model in Comprehensive Meta-Analysis software (CMA 4.0) was used for meta-analysis, along with heterogeneity testing, sensitivity analysis, and publication bias assessment. Finally, a moderating analysis was conducted based on the extracted data.

**Results:**

A total of 41 studies involving 9693 participants and reporting 48 dropout rates were included. The mean dropout rate in the intervention group after 18 studies was 22% (95% CI 0.13‐0.36), which was lower than the control group’s 26% (95% CI 0.16‐0.39). High heterogeneity was observed between studies (Q=396.18, *df*=17, *P*<.001, I²=96%), and moderating analysis revealed that high heterogeneity in dropout rates was associated with four variables across three major characteristics: (1) participant demographic characteristics: employment rate; (2) participant clinical characteristics: baseline clinical diagnosis and baseline drug use type; and (3) intervention characteristics: intervention frequency. In the 30 studies with the longest follow-up period in the intervention group, the dropout rate was 28.2% (95% CI 0.19‐0.39), comparable to the control group’s 27.8% (95% CI 0.20‐0.37). Extremely high variability was observed between studies (Q=1293.13, *df*=29, *P*<.001, I²=98%), and moderating analysis showed that high heterogeneity in dropout rates was associated with 4 variables across three major characteristics: (1) participant demographic characteristics: single individuals; (2) participant clinical characteristics: baseline medication frequency; and (3) treatment characteristics: recruitment method and the degree of digitalization. Additionally, publication bias assessment and sensitivity analysis supported the robustness of the study results.

**Conclusions:**

This study explored the impact of digital psychosocial interventions on treatment adherence among adult illicit drug users, revealing complex factors affecting dropout rates through mediation analysis. These findings not only emphasize the necessity of further research but also provide important evidence for developing precision interventions, holding significant implications for both theory and clinical practice.

## Introduction

The global issue of illicit drug use has worsened, with 292 million users in 2022, a 20% increase over the past decade [1]. Cannabis is the most widely used illicit drug (228 million), followed by opioids (60 million), cocaine (23 million), and others [1]. Illicit drug users face various psychological and physiological problems, including mental disorders, cognitive deficits, cardiovascular dysfunction, and blood-borne infections. The social burden is also high, due to links with crime, violence, and sexual abuse [2]. Treatment is urgently needed, but globally, only about 10% of users receive treatment, a decline since 2015 [1].

Traditional face-to-face psychosocial treatments remain important for illicit drug users but often fail to meet the needs of most patients due to time, location, and social stigma [3]. The COVID-19 pandemic accelerated the development of telehealth [4] and pushed digital interventions from early simple interactions to more complex forms [5]. Modern digital interventions can provide multiple interaction methods via smart devices, such as apps, websites, email, text messages, video, audio, and computer programs. They overcome the limitations of traditional treatments and are valued for their flexibility and cost-effectiveness [6-10], better meeting personalized needs and improving treatment engagement [11]. Meta-analyses show that digital interventions are effective across different populations of illicit drug users [12-14].

However, dropout rates are particularly prominent in digital interventions [15-17]. Meta-analyses indicate that about one-third of individuals with substance use disorders fail to complete treatment [18] and only 48% of early dropouts seek help again [19], significantly increasing the risk of adverse outcomes [20,21]. Methodologically, the relatively high dropout rate limits the completeness of research findings, affecting the validity of results and the interpretation of treatment effects [22]. To improve the accuracy, this study clearly distinguishes three key concepts: engagement refers to behavioral involvement during use [23]; adherence reflects the alignment between actual behavior and intervention expectations [24]; while the dropout rate in this study is strictly defined as participants leaving, being lost to follow-up, or stopping participation before the outcome assessment for any reason. This conceptual clarification both distinguishes commonly confused terms and provides a methodological basis for enhancing the effectiveness of digital interventions, with important clinical implications.

Although the dropout rate is an important outcome indicator of intervention efficacy [25], few studies have examined dropout rates among illicit drug users in digital interventions. A meta-analysis published in 2017 was the first to evaluate internet-based interventions in reducing illicit substance use after treatment and follow-up, but dropout rate was not the focus [12]. Moreover, existing research lacks systematic examination of clinical factors and intervention design, as well as dynamic assessment of dropout patterns at different time points [26-28], directly limiting the optimization of targeted intervention strategies.

Based on current research, this study aims to address the gap in dropout rate research in digital interventions. The study compared average dropout rates between the digital intervention and control groups to assess treatment retention under different experimental conditions. It also analyzed how variables at posttreatment and the longest follow-up time points affected dropout rates in the intervention group to support personalized intervention design for different research stages. These findings are important for advancing academic research and expanding clinical applications [29].

## Methods

### Protocol Registration

This study strictly adheres to the guidelines of the Cochrane Handbook for Interventions [30] and is reported according to the PRISMA (Preferred Reporting Items for Systematic Reviews and Meta-Analyses) 2020 guidelines [31] (the complete PRISMA checklist is available in Checklist 1). The research protocol has been registered in the PROSPERO system: CRD42024534389.

### Search Strategy

To comprehensively and systematically collect relevant literature, this study searched five major databases up to August 27, 2025, including Web of Science, PubMed, PsycINFO, Embase, and the Cochrane Controlled Trials Register. The search strategy combined controlled vocabulary (eg, MeSH terms) and free-text keywords using Boolean operators (“AND” and “OR”). The main search terms included the following: (“digital intervention” OR “internet intervention” OR “e-health” OR “m-health”) AND (“drug abuse” OR “substance use disorder” OR “illicit drugs”) AND (“psychotherapy” OR “psychoeducation” OR “psychodynamic”) AND (“randomized controlled trial” OR “single blind procedure” OR “random sample”). The complete search strategy for each database is provided in Multimedia Appendix 1.

### Inclusion and Exclusion Criteria

Inclusion criteria were as follows: (1) Individuals aged 18 years and above with illicit drug use behavior. Illicit drugs refer to controlled substances used for nonmedical or nonscientific purposes, including but not limited to cannabis, cocaine, amphetamines, and opioids [1]. (2) Digital psychosocial intervention is the primary treatment. Operationally defined as structured psychological intervention primarily delivered through digital platforms, including mobile applications, web-based programs, or digital communication tools, with or without minimal human support. (3) The article must report sample size and dropout rates. (4) Randomized controlled trials. Exclusion criteria were as follows: (1) treatment involving only face-to-face therapy. (2) mixed samples with insufficient proportion of illicit drug users (less than 80%) or without independent subgroup data (eg, alcohol and tobacco users). (3) non-English studies. (4) unpublished reports, study protocols, meta-analyses, reviews, doctoral theses, or other gray literature.

To ensure the accuracy of literature screening, a dual-screening process was adopted. First, two researchers independently screened the titles and abstracts of retrieved literature to exclude those clearly not meeting inclusion criteria. Subsequently, the full texts of the literature were reviewed for further evaluation. Finally, manual searches were conducted on the reference lists of included studies and related reviews to identify additional studies meeting inclusion criteria. Any disagreements were resolved through discussion.

### Select Variables and Data Extraction

#### Outcome Variable

This study uses the dropout rate from randomized controlled trials as the primary outcome measure. Considering that the influencing factors at different treatment stages may vary [3,32-34], the dropout rate data of the intervention and control groups at the end of treatment and at the longest follow-up time were extracted separately.

#### Moderator Variables

Previous studies have explored the factors influencing dropout among illegal drug users [35], but due to differences in confounding variable control methods and insufficient understanding of the complexity of predictive factors, the results have been inconsistent [32]. Withdrawal from treatment is a dynamic process, and its mechanisms involve complex interactions of multiple factors [36]. It is difficult to fully explain the complexity of single-variable analysis [22]. Therefore, this study refers to previous research [37] and selects multidimensional variables (Table 1): (1) Demographic characteristics of participants: most studies emphasize the role of patient-related variables in predicting dropout [38,39], and investigating individual differences (such as age, gender, race, digital literacy, etc.) is crucial for developing treatment interventions for specific populations [16]. (2) Baseline clinical characteristics of participants, including the type of illegal drug use, medication patterns, frequency of use, duration of use, and comorbid conditions. Different drugs may have differentiated effects on dropout rates due to their unique pharmacological mechanisms and withdrawal characteristics [40]. Additionally, the presence of comorbid mental disorders may exacerbate the likelihood of treatment interruption [41], which also needs to be considered. (3) Therapist characteristics: the therapeutic orientation and experience level of therapists may be related to patient adherence [42]. Compared to busy clinic staff, full-time therapists are more likely to invest time and effort to retain and reengage patients who have discontinued treatment [32]. (4) Treatment characteristics: referring to the framework proposed by Derubeis et al [43], which focuses on all factors that improve treatment and particularly on the relationship between treatment factors and outcomes. For example, this study extracted personalized feedback, real-time interaction, and therapeutic alliance. The optimization of these modifiable operational variables can directly enhance intervention effectiveness and improve patient treatment adherence [44].

**Table 1.** Predictor variables.

Predictor category	Variable category	Variable	Data note
Demographic characteristics of participants	Continuous variable	Year	Publication year
		N^a^	Number of participants
		Age	Mean years
		Female	Percentage
		White	Percentage
		African American	Percentage
		Education	≦High school degree (%)
		Employed^b^	Percentage
		Unemployed^b^	Percentage
		Single/never married	Percentage
		Currently single	Percentage
		Married/living together	Percentage
	Classified variable	Developed country^c^	Y^d^^,^ N^e^
		Low income	Y, NR^f^
Baseline clinical characteristics of participants	Continuous variable	Diagnostic	Percentage
		Use quantity-pre	Mean percentage of substance use quantity in the past 30 days
		Use frequency-pre	Mean percentage of substance use frequency in the past 30 days
		Use length-pre	Mean length of substance use in years at intake
		Abstinence	Percentage
	Classified variable	Inclusion criteria	Diagnostic and Statistical Manual (DSM) diagnosis, Other
		Comorbid HIV	Y, N
		Primary drug use	Cocaine, Opioids, Cannabis, ATS^g^^,^ Other
Therapist characteristics	Classified variable	Master	Y, NR
		Relevant experience	Y, NR
		Train	Y, NR
		Supervision	Y, NR
Treatment characteristics	Continuous variable	Session	Number of weekly sessions
		Intervention duration	Number of weeks
		The longest follow-up	Number of weeks
	Classified variable	Recruitment	Website, Clinic, Community, Campus, Multiple
		Compensation mode	Gift certificate, USD^h^
		Compensation^i^	Stepped^j^, NR
		Measurement	Self-report, Toxicology, Both
		Toxicology	Y, N
		Guidance	Guided, Unguided
		Personalized feedback/intervention	Y, NR
		Real-time interaction	Y, NR
		Setting	Anywhere, Laboratory
		Delivery	Computer, Telephone
		Digital media	App, Website
		Digital presentation mode	Video, Virtual character
		Fully digital	Y, N, NR
		Assessing digital quality	Y, NR

a N: Number.

b “Employed” and “Unemployed”: Not complementary, they were extracted separately from different studies. We extracted only based on the study reports and did not perform back-extrapolation calculations.

c Developed country: According to the World Health Organization.

d Y: Yes.

e N: No.

f NR: Not reported.

g ATS: Amphetamine-type stimulants.

h USD: Use USD as experimental compensation.

i Compensation: Refers to the monetary or nonmonetary rewards provided to study participants for their time and effort.

j Stepped: Refers to a structured payment approach where participants receive partial rewards at different stages (eg, time-based or task-completion).

### Data Extraction

Two researchers independently extracted data using a predesigned data extraction form. Disagreements were resolved through discussion or consultation with a third researcher. This data extraction form has been piloted in some studies and adjusted according to the recommendations and structured framework of the GRADE manual. For articles that met the inclusion criteria but lacked important data, we contacted the corresponding author via email, and studies that could not provide sufficient data to calculate effect sizes were excluded.

### Quality Assessment

To assess the bias risk of the included studies, two researchers independently scored each study in five aspects using the revised Cochrane Risk of Bias tool ROB 2.0 [45]: randomization process, deviations from intended interventions, missing outcome data, measurement of the outcome, and selection of the reported result. Any disagreements were resolved through discussion.

### Statistical Analysis and Software

We used Comprehensive Meta-Analysis software (CMA 4.0) to synthesize dropout rates across studies [46]. For each trial, dropout counts and total sample sizes were extracted separately for the intervention and control groups, from which group-specific dropout proportions were calculated. To stabilize variances and account for the bounded nature of proportions, these proportions were transformed into logit event rates with corresponding standard errors, which served as the primary effect size metric. Pooled estimates were calculated separately for intervention and control groups and subsequently back-transformed into raw proportions and expressed as percentages for interpretability, an approach that has been widely applied in meta-analyses of proportion-type outcomes [47]. Subsequently, between-study heterogeneity was examined using the Q statistic [48,49] and quantified with the I² statistic [50]. Given the significant heterogeneity among included studies in outcome measures and moderators [51,52], all analyses were conducted under a random-effects model [53]. Publication bias was assessed using funnel plots, Egger’s, Duval and Tweedie’s trim and fill, and Classic fail-safe N tests [54], while sensitivity analyses were conducted to evaluate the robustness of the results. To explore potential influencing factors, meta-regression and subgroup analyses were further employed to examine the association between moderators in the intervention group and dropout rate.

## Results

### Characteristics of the Included Studies

**Figure 1.**
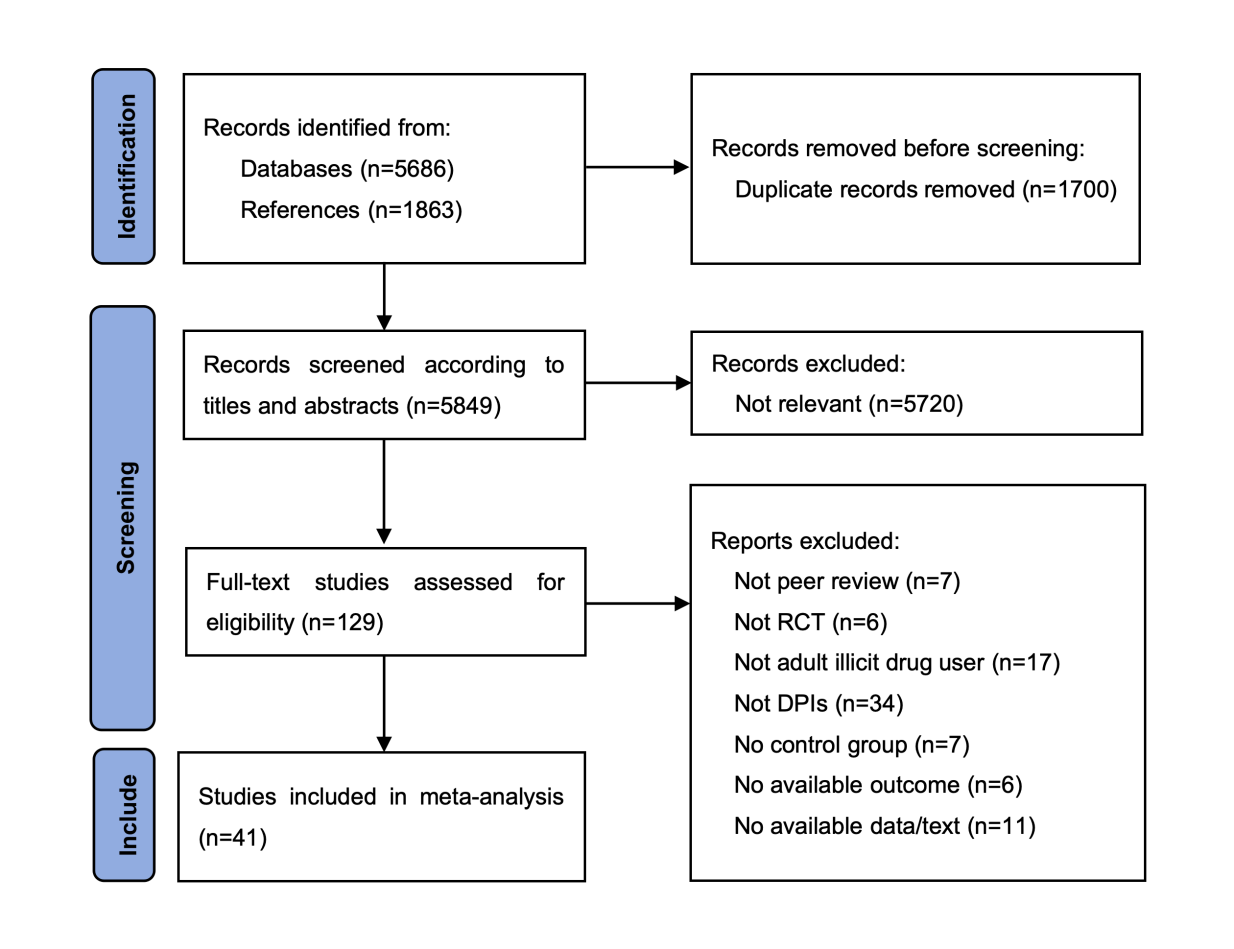
PRISMA flow diagram of study search and selection. DPI: Digital psychosocial intervention; PRISMA: Preferred Reporting Items for Systematic reviews and Meta-Analyses; RCT: randomized controlled trial.

After screening relevant articles based on predefined inclusion and exclusion criteria, a total of 41 studies were finally included (see Figure 1), involving 9693 participants with an age range of 19 to 50 years. The selection characteristics of the included studies are shown in Table 2. The studies included 82 intervention groups, with a total of 48 dropout rate data points, including 18 posttreatment dropout rates and 30 follow-up dropout rates, showing different data results between the two measurement points.

**Table 2.** Selected characteristics of included studies.

Author (year)	Country	N^a^	Recruitment	Primary substance	Intervention type	Age, M (SD)	F (%)	Intervention duration (wk）	Sessions	The longest follow-up (wk)
Aharonovich (2012)[55]	USA	40	Clinic	Cocaine/crack (75.8%)	MI^b^+BI^c^	45.5 (6.6)	24.2	8	7.00	NR^d^
Aharonovich (2017a)[56]	USA	240	Clinic	Any	MI+BI	46.5 (9.3)	16.3	8.57	7.00	48
Aharonovich (2017b)[57]	USA	47	Multiple	Crack (91.49%)	MI+BI	50.9 (7.0)	23.4	8.57	7.00	NR
Baumgartner (2021)[58]	Switzerland, Austria, Germany, Other (0.7%)	575	Website	Cannabis (100%)	CBT^e^+MI+BI	28.3 (7.9)	29.4	6	NR	12
Blow (2017)[59]	USA	780	Clinic	Cannabis (91.1%)	MI	31.2 (10.9)	55.5	1	1.00	12
Bonar (2022)[60]	USA	149	Website	Cannabis (100%)	CBT+MI	21 (2.2)	55.7	8	7.00	24
Bonar (2023)[61]	USA	58	Clinic	Cannabis (100%)	MI	21.5 (2.4)	65.5	4	7.00	12
Brooks (2010)[62]	USA	28	Clinic	Cocaine	CRA^f^	43.1 (9.2)	55	8	3.00	10
Buckner (2020)[63]	USA	63	Campus	cannabis	BI	19.1 (1.5)	84.1	64	1	2
Budney (2011)[64]	USA	38	Community	Cannabis (100%)	MET^g^+ CBT+CM^h^	32.8 (9.7)	47.1	12	1.00	NR
Budney (2015)[65]	USA	75	Multiple	Cannabis (100%)	MET+ CBT+CM	35.9 (10.5)	43	12	2.00	36
Campbell (2014)[66]	USA	507	Clinic	Any	CRA+CM	34.9 (10.9)	37.9	12	4.00	24
Carroll (2014)[67]	USA	101	Clinic	Cocaine (100%)	CBT	41.9 (9.6)	60.4	8	7.00	24
Chopra (2009)[68]	USA	120	Community	Opioid (100%)	CRA+CM	31.8 (10.5)	42.5	12	3.00	NR
Christensen (2014)[69]	USA	170	Multiple	Opioid (100%)	CRA+CM	34.3 (10.8)	45.9	12	3.00	NR
Christoff (2015)[25]	Brazil	458	Campus	Any	MI	24 (5.4)	7	0.14	1.00	12
Chun-Hung (2023)[70]	Taiwan, China	99	Clinic	ATS^i^ (100%)	MBRP^j^	37 (10.4)	18.2	NR	4.20	24
Conner (2024)[71]	Canada, USA	781	Campus	Cannabis	BI	21.7 (2.8)	39.7	0.14	1	4
Coronado-Montoya (2025)[72]	Canada	101	Clinic	Cannabis (100%)	CBT+MI	25.2 (3.9)	18.8	6	1	18
Dunn (2017)[73]	USA	76	Clinic	Opioid (100%)	PE^k^	39.9 (12.7)	40.8	1	1.00	12
Elliott (2014)[74]	USA	162	Campus	Cannabis (100%)	PE	19.3 (1.2)	52	NR	NR	4
Glasner (2022)[75]	USA	54	Multiple	Opioid (50%), ATS (50%)	CBT	47.7 (8.2)	20	12	7.00	NR
Gryczynski (2015)[76]	USA	360	Clinic	Any	MI	36.2 (14.6)	46	NR	NR	48
Gryczynski (2016)[77]	USA	80	Community	Any	MI	35 (13)	53	1	1.00	24
Gustafson (2024)[78]	USA	414	Clinic	Opioid	PE+BI+MI	37.2 (10.0)	45.2	64	NR	32
Ingersoll (2011)[79]	USA	56	Community	Crack cocaine (100%)	PE	45 (6.4)	51.9	8	0.75	24
Maricich (2021)[80]	USA	170	Multiple	Opioid (100%)	CRA	32.9 (9.8)	45.9	12	2.50	NR
Marsch (2014)[81]	USA	160	Community	Opioid (100%)	CRA+CBT	40.7 (9.8)	25	48	0.54	NR
Moore (2019)[82]	USA	82	Clinic	Any	CBT	42.4 (10.9)	40.2	12	7.00	12
Olthof (2023)[83]	Netherlands	378	Website	Cannabis (100%)	CBT+MI	27.5 (8.5)	30.7	NR	NR	24
Ondersma (2007)[84]	USA	107	Clinic	Any	MI	25.1 (5.6)	100	1	1.00	24
Ondersma (2014)[85]	USA	143	Clinic	Any	MI	26.6 (6)	100.0	1	1.00	24
Schaub (2019)[86]	Switzerland	311	Website	Cannabis (100%)	PE+CBT+MI	33.1 (7.6)	27	6	1.50	24
Schaub (2015)[87]	Switzerland	308	Multiple	Cannabis (100%)	CBT+MI	29.8 (10)	24.7	6	NR	12
Schwartz (2014)[88]	USA	360	Community	Cannabis (88%)	BI	36.1 (14.6)	46	1	1.00	12
Shi (2019)[89]	USA	20	Community	Opioid (100%)	CBT	40.5 (12.2)	40	12	6.88	NR
Sinadinovic (2020)[90]	Sweden	303	Website	Cannabis (100%)	PE+CBT+MI	27.4 (7.2)	32.7	6	1.50	12
Tait (2015)[91]	Australia	160	Multiple	ATS (100%)	CBT+MI	22.4 (6.3)	24	NR	NR	24
Tossmann (2011)[92]	Germany	1292	Website	Cannabis (100%)	SFBT^l^	24.7 (6.8)	29.5	7.14	NR	12
Walukevich-Dienst (2019)[93]	USA	227	Campus	Cannabis (100%)	PE	19.8 (1.4)	77	NR	NR	4
Xu (2021)[94]	China	40	Community	ATS (>90%)	PE+ST^m^	46.1 (9.9)	22.5	NR	1.00	24

a N: Number of participants.

b MI: Motivational interviewing.

c BI: Brief intervention.

d NR: Not reported.

e CBT: Cognitive behavior therapy.

f CRA: Community reinforcement approach.

g MET: Motivational enhancement therapy.

h CM: Contingency management.

i ATS: Amphetamine-type stimulants.

j MBRP: Mindfulness-based relapse prevention.

k PE: Psychoeducation.

l SFBT: Solution-focused brief therapy.

m ST: Support.

### Risk of Bias Assessment

The risk of bias in the included studies was assessed using the Cochrane Risk of Bias tool (ROB 2.0). Detailed results and percentage plots are presented in Multimedia Appendix 2. The results showed that approximately 90% of the included studies had a low risk in terms of the randomization process (D1), measurement of the outcome (D4), and selection of the reported result (D5). Approximately 55% of the included studies had some concerns about deviations from intended intervention (D2). About 50% of the studies had a high risk of missing outcome data (D3), which is a key focus of our research.

### Meta-Analysis Results

#### Posttreatment

**Figure 2.**
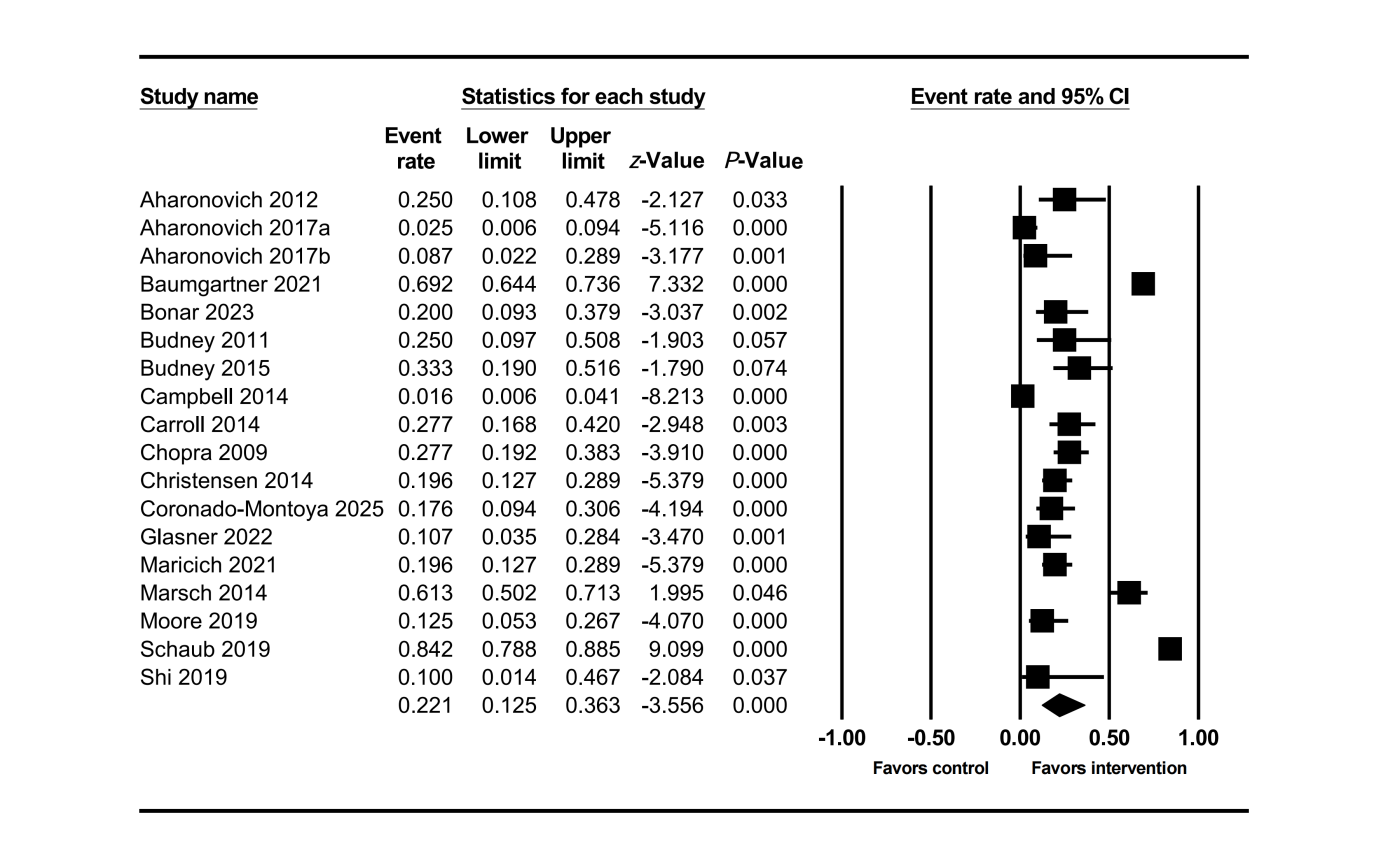
Forest plot of dropout rate in the intervention group at posttreatment [55-58,61,64-69,72,75,80-82,86,89].

An analysis of 18 studies was conducted using a random-effects model. The main effect results (Figure 2) showed that the mean dropout rate in the intervention group was 22% (95% CI 0.13‐0.36), lower than that in the control group of 26% (95% CI 0.16‐0.39) [51]. However, heterogeneity testing indicated high variability among the studies (Q=396.18, *df*=17, *P*<.001; I²=96%). Further analysis revealed that the variance of the true effect size reached 2.02 (logit units) with a standard deviation of 1.42 (logit units).

Meta-regression and subgroup analysis revealed that this extreme variability was primarily due to four variables among three categories (Table 3): (1) Participant demographic characteristics: The proportion with employment rate showed a weak positive correlation with dropout rate (OR 1.04, 95% CI 1.00‐1.07; *P*=.03). (2) Participant clinical characteristics: Participants with baseline clinical diagnoses showed a significant positive correlation with dropout rate (odds ratio [OR] 1.03, 95% CI 1.01‐1.06; *P*=.01). The dropout rate for those using cocaine as the baseline primary medication (OR 1.96, 95% CI 0.31‐12.57; *P*=.48) was significantly higher than that for those using cannabis and opioid medications. (3) Intervention characteristics: Intervention frequency showed a significant negative correlation with dropout rate (OR 0.77, 95% CI 0.60‐0.99; *P*=.04). The other 27 factors showed no significant correlation with dropout rate.

**Table 3.** Meta-regressions and subgroup analysis in the intervention group at posttreatment.

Predictor category	Predictor/Predictor value	Studies	Coefficient	Standard error	Dropout (95% CI)	z-value	2-sided *P* value
Demographic characteristics of participants	Employed	6	0.0348	0.0159	0.0036 to 0.0661	2.19	.0288
Baseline clinical characteristics of participants	Diagnostic	12	0.0305	0.0125	0.0060 to 0.0549	2.44	.0145
	Primary drug use	17					.0190
	Cocaine	3	0.6738	0.9478	−1.1838 to 2.5314	0.71	.4771
	Opioid	5	−0.2639	0.8303	−1.8912 to 1.3634	−0.32	.7506
	Cannabis	5	−0.7448	0.5838	−1.8889 to 0.3994	−1.28	.2020
	Other	4	−2.2799	0.9056	−4.0548 to −0.5050	−2.52	.0118
Treatment characteristics	Session	17	−0.2609	0.1266	-0.5090 to −0.0127	−2.06	.0394

**Figure 3.**
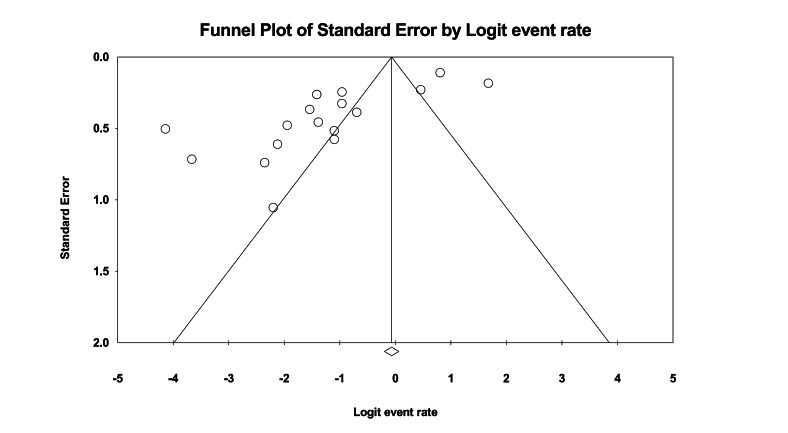
The funnel plot for dropout rate in the intervention group at posttreatment.

**Figure 4.**
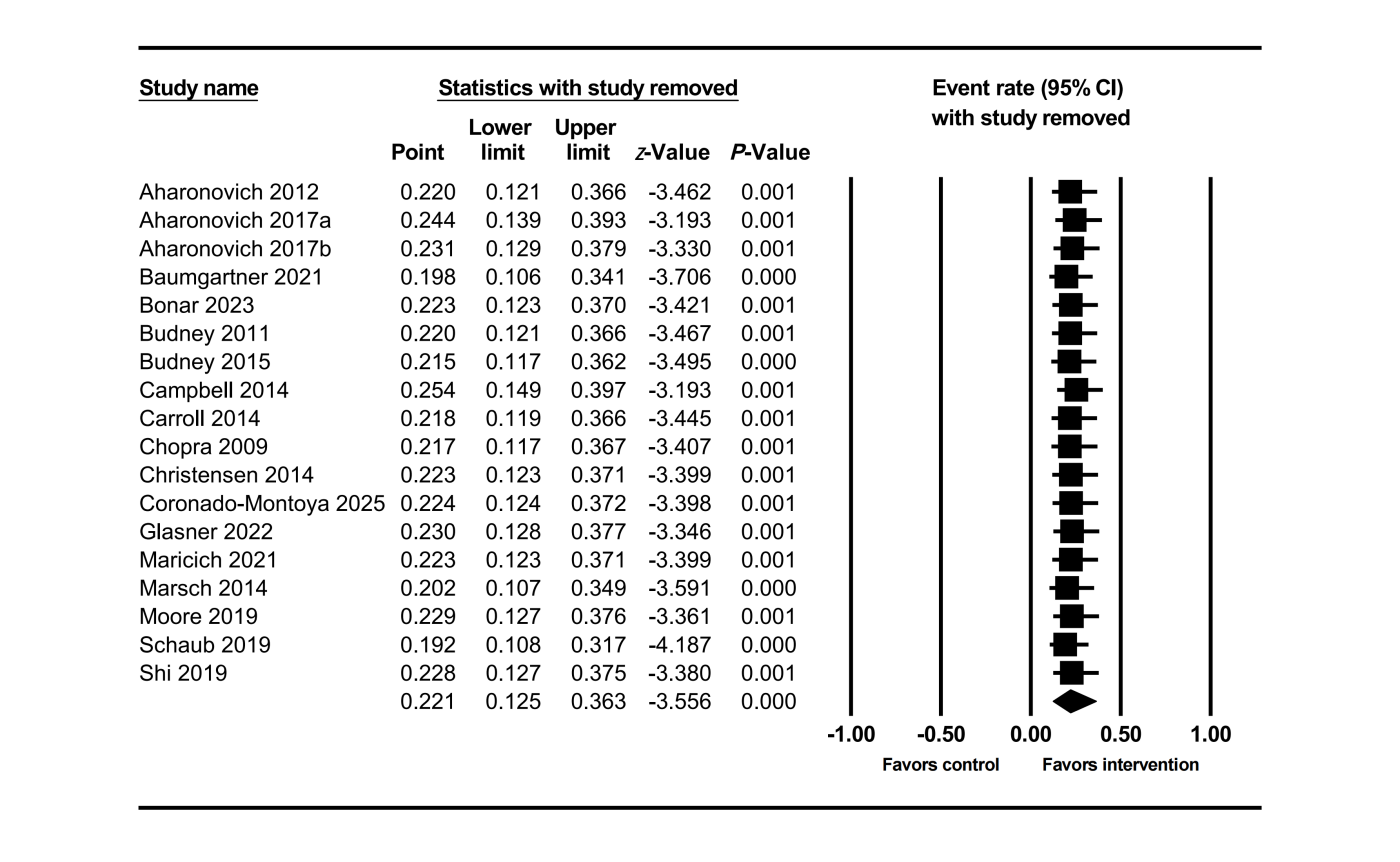
Sensitivity analysis for dropout rate in the intervention group at posttreatment [55-58,61,64-69,72,75,80-82,86,89].

The funnel plot showed some studies beyond the expected range (Figure 3), suggesting the presence of studies with extreme dropout rates. Combined with Egger’s test results (*P*<.001), this further confirmed the presence of publication bias. After trimming the 5 missing studies on the right side, the effect size was adjusted from 22% to 33%, still not crossing the clinical threshold. Further leave-one-out analysis showed that 366 unpublished studies would need to be included to make the current result statistically insignificant. Overall, the results indicate that despite publication bias, the adjusted effect size did not exceed the clinical threshold and the leave-one-out number was high, supporting the stability of the study conclusions. Sensitivity analysis also showed (Figure 4) that removing any single study would not change the overall trend.

### The Longest Follow-Up

**Figure 5.**
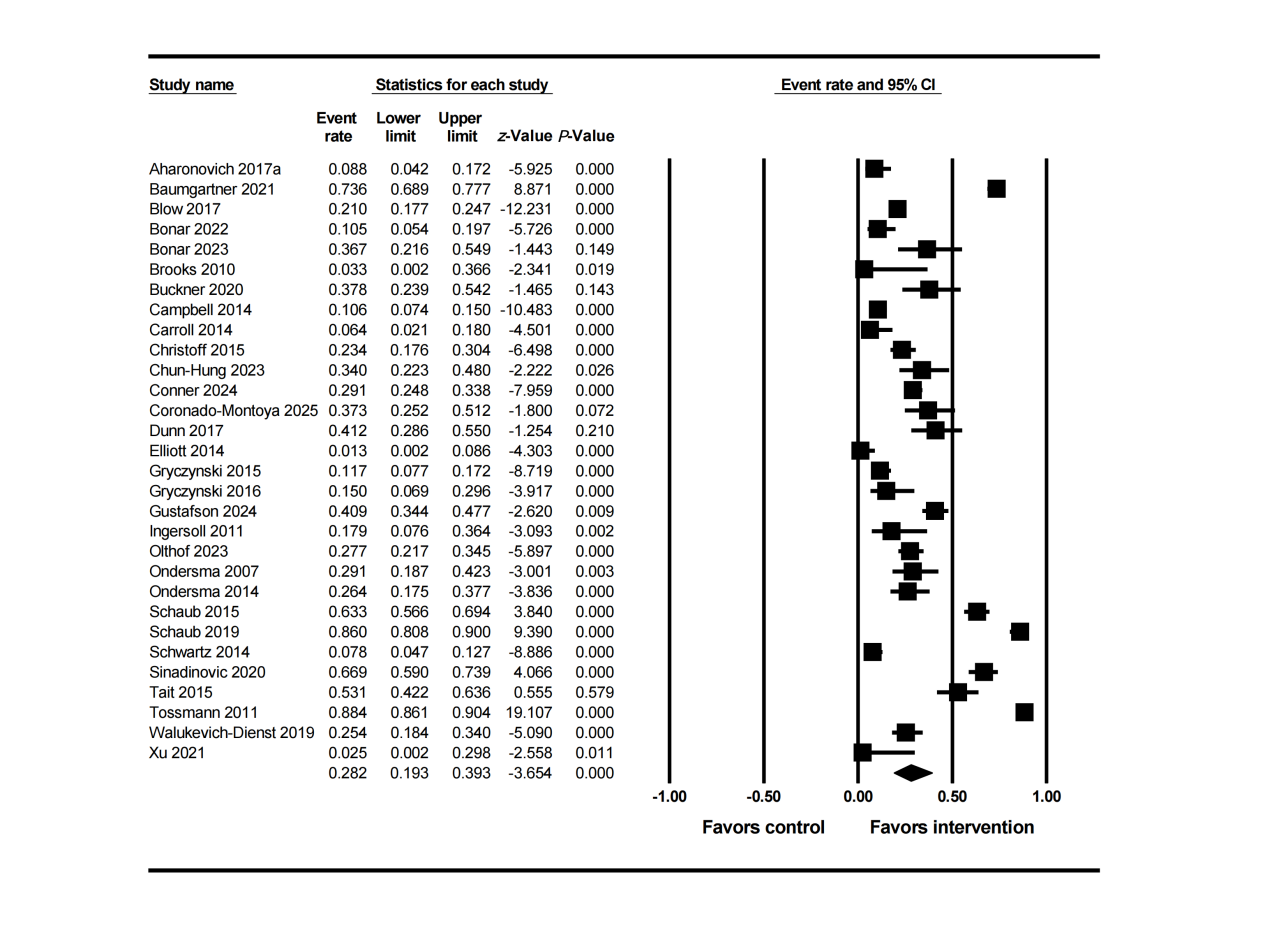
Forest plot of dropout rate in the intervention group at the longest follow-up [25,56,58-63,66,67,70-74,76-79,83-88,90-94].

Follow-up analysis of the intervention group was based on 30 studies, with an average dropout rate of 28.2% (95% CI 0.19‐0.39) (Figure 5), while the rate in the control group was 27.8% (95% CI 0.20‐0.37). However, heterogeneity testing again indicated high variability among the studies (Q=1293.13, *df*=29, *P*=.000, I²=98%). Further analysis revealed that the variance of the true effect size reached 1.79 (logit units) with a standard deviation of 1.34 (logit units).

Meta-regression analysis and subgroup analysis (Table 4) revealed that this extreme variability is primarily due to 4 variables among three types of characteristics: (1) participant characteristics: dropout rate showed a negative correlation with single status (OR 0.95, 95% CI 0.91‐0.99; *P*=.01); (2) clinical characteristics: significantly positive correlation with baseline medication frequency (OR 1.18, 95% CI 1.05‐1.32; *P*=.004); (3) intervention characteristics: participants recruited via website showed a positive correlation with dropout rate (OR 5.74, 95% CI 1.85‐17.76; *P*=.002), while participants recruited via campus showed a negative correlation with dropout rate (OR 0.28, 95% CI 0.12‐0.66; *P*=.003); The association between the degree of digitalization and dropout rates varied depending on whether studies with unreported digitalization status (not reported [NR] group) were included. When all studies, including the NR group, were analyzed, the overall model reached statistical significance (Q=28.13, *df*=2, *P*<.001), with the NR group showing a strongly significant negative effect (OR 0.16, 95% CI 0.06‐0.41; *P*<.001). However, when the NR group was excluded and only studies explicitly reporting “fully digital” or “partially digital” were considered, the results were not statistically significant (Q=0.24, *P*=.62). The other 32 factors showed no significant correlation with dropout rate.

**Table 4.** Meta-regression and subgroup analysis in the intervention group at the longest follow-up.

Predictor category	Predictor/Predictor value	Studies	Coefficient	Standard error	Dropout (95% CI)	z-value	2-sided *P* value
Demographic characteristics of participants	Currently single	10	−0.0528	0.0214	−0.0947 to −0.0108	−2.47	.0136
Baseline clinical characteristics of participants	Use frequency-pre	10	0.1657	0.0576	0.0528 to 0.2786	2.88	.0040
Treatment characteristics	Recruitment	28					
	Website	6	1.7478	0.5762	0.6168 to 2.8770	3.03	.0024
	Clinic	12	0.0973	0.5204	−0.9225 to 1.1172	0.19	.8516
	Campus	5	−1.2797	0.4371	−2.1365 to −0.4230	−2.93	.0034
	Community	5	−0.8413	0.6384	−2.0924 to 0.4099	−1.32	.1875
	Fully digital	30					
	No	4	0.5442	0.4530	−0.3437 to 1.4320	1.20	.2297
	Yes	3	0.2858	0.6540	−0.9960 to 1.5676	0.44	.6621
	Not reported	23	−1.8401	0.4882	−2.7970 to −0.8831	−3.77	.0002

**Figure 6.**
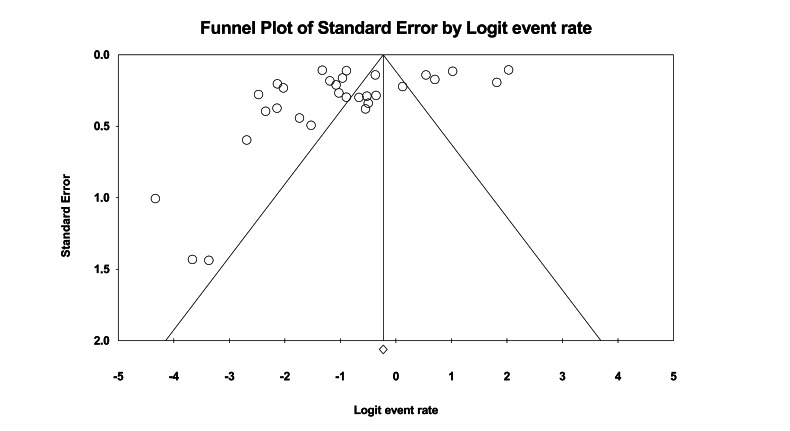
The funnel plot for dropout rate in the intervention group at the longest follow-up.

**Figure 7.**
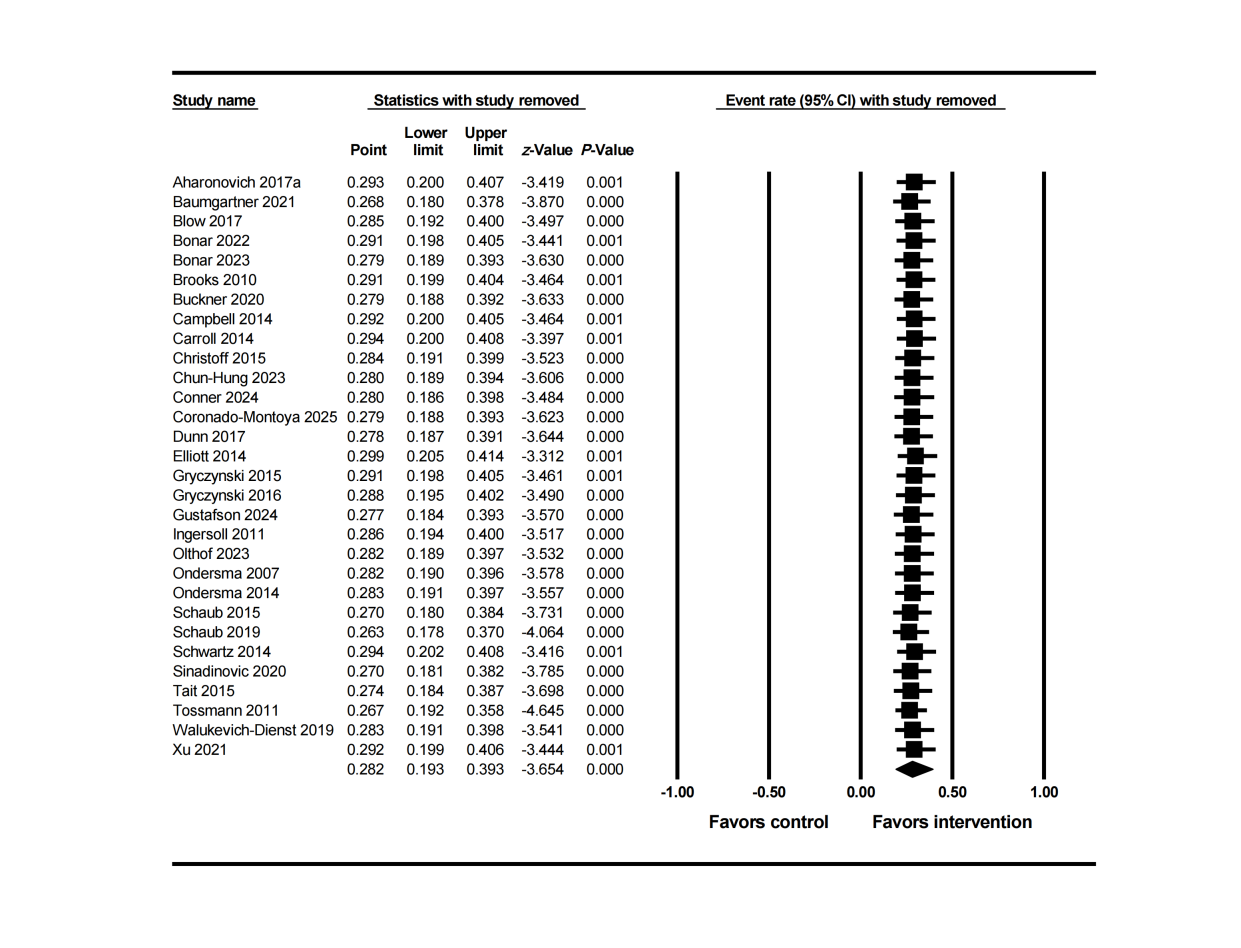
Sensitivity analysis for dropout rate in the intervention group at the longest follow-up [25,56,58-63,66,67,70-74,76-79,83-88,90-94].

The funnel plot showed some studies beyond the expected range (see Figure 6). Combined with Egger test results (*P*=.023), publication bias was further confirmed. After trimming the six missing studies on the right side, the effect size changed from 28% to 37% after correction, without crossing the clinical threshold. Further leave-one-out sensitivity analysis showed that 1244 unpublished studies would need to be included to make the current results statistically insignificant, supporting the stability of the research conclusion. Meanwhile, sensitivity analysis (see Figure 7) indicated that the results of this study were robust and not dependent on individual studies.

## Discussion

### Principal Findings

This meta-analysis systematically evaluated the treatment retention effect of digital psychosocial interventions among adult illicit drug users. The pooled dropout rate was 22%, slightly lower than the approximately 30% reported for face-to-face psychosocial interventions [37], suggesting potential advantages of digital formats for treatment retention. Nevertheless, the substantial heterogeneity across studies limits the generalizability of these findings. Dropout rates also varied across settings and populations. For instance, adults with co-occurring severe mental disorders and substance use had an average dropout of 27% [95], whereas clinical samples of opioid users showed rates as high as 41% [73]. Beyond dropout, adherence constitutes another key indicator of engagement, with evidence showing that participants completed, on average, 60% of digital intervention modules, and only about half finished the full program [96]. Taken together, these results underscore the importance of considering both dropout and adherence when evaluating intervention effectiveness. Building on this, our moderator analyses further revealed complex interactive effects. To ensure clarity, we retained the classification system established during data extraction, presenting results separately across four major categories of characteristics as well as between short-term and longest intervention stages.

At the posttreatment stage, dropout was significantly influenced by participants’ demographic, intervention, and clinical characteristics. Regarding demographics, unemployment did not predict dropout, whereas higher employment was unexpectedly associated with greater attrition. This suggests that unstable or high-intensity work may interfere with regular participation. In addition, the short-term income from employment may reduce some patients’ motivation for treatment, especially when symptoms temporarily improve, leading them to discontinue prematurely due to “feeling better” [97]. For intervention characteristics, intervention frequency showed a negative correlation with dropout, indicating that more frequent contact may help consolidate behavior change, strengthen the therapeutic alliance, and enhance commitment [98-100]. Future studies should explore the optimal intervention frequency under different conditions [101], balancing treatment intensity with patient burden [102].

The results of baseline clinical characteristics indicated that both baseline clinical diagnosis and baseline cocaine use were significantly positively associated with dropout rates. Specifically, patients with a clear baseline diagnosis were at greater risk of dropout due to challenges such as dependency, withdrawal symptoms, and impaired cognitive or emotional functioning [42]. For this population, the integration of adjunctive pharmacological or behavioral therapies is recommended to reduce dropout [103]. Furthermore, consistent with previous findings [32], participants with baseline cocaine use were more likely to discontinue treatment. Cocaine use disorder is often closely linked to impulsive behavior and diminished adherence [37]. These substance-specific risks highlight the importance of developing differentiated intervention strategies tailored to distinct types of substance use in future research [104]. Nevertheless, the small sample size of drug-use subgroups (k≤5) remains a limitation, which could be addressed through multi-institutional collaborations to expand subgroup samples.

During the longest follow-up, dropout was significantly influenced by demographic, clinical, and intervention characteristics. In demographics, a higher proportion of single participants was linked to lower dropout. This may be related to reduced drug exposure in family environments [105-107]. In addition, single participants with low social support were more likely to continue seeking health information online. Future research could involve non–drug-using significant others in monitoring the intervention process and integrate peer support modules [108]. In clinical characteristics, participants with higher baseline drug use frequency faced markedly greater dropout risk. This finding is consistent with recent studies [109]. For this high-risk group, we recommend the implementation of multistage intensive intervention programs [110], together with the development of immediate-response modules (eg, crisis management tools, real-time consultation functions) to reduce early dropout [81].

In terms of intervention characteristics, participants recruited through websites exhibited higher dropout rates, whereas those recruited from campus showed lower dropout rates. This may be explained by the lack of intensive treatment services typically provided in clinical settings, as well as the relative stability of campus environments [6,111]. Based on this finding, we recommend adopting a mixed online–offline recruitment strategy [112]. In addition, intervention content should be optimized for online recruits [113], including simplifying operational procedures, providing regular reminders, and offering personalized feedback. The study also analyzed the association between the degree of digitalization and dropout rates. During data processing, studies that did not report their digitalization status (23/30, 77%) were categorized separately as a “Not reported” group for analysis rather than being directly excluded. The analysis revealed a significant association: compared to the nonsignificant negative correlation between fully digital interventions and dropout rates, interventions with unreported digitalization status showed a significant negative correlation, while non-fully digital interventions demonstrated a significant positive correlation with dropout rates. However, the reliability of these subgroup comparisons is constrained by the prevalent issue of poorly reported data. When we excluded the “Not reported” studies and repeated the analysis, no significant differences were found between fully digital and partially digital interventions. This suggests that the initial findings were likely confounded by nonrandom reporting bias rather than reflecting true effects, making definitive evaluation difficult. Therefore, these results primarily highlight the urgent need for future research to standardize the reporting of specific digital intervention details in order to more reliably explore the role of digitalization degree and human support in improving retention rates [114].

### Research Significance

This study systematically evaluated the dropout rate and its predictive factors among adult illicit drug users in digital psychological interventions, thereby addressing a critical research gap in the field. Unlike previous studies that primarily focused on demographic characteristics, this analysis incorporated multidimensional predictive variables—including clinical features, therapist-related factors, and intervention characteristics—to establish a more systematic theoretical framework. The identification of eight key predictive factors provides valuable insights for personalized interventions, guiding the development of tailored digital tools for patients at high risk of dropout. Optimization strategies derived from this evidence are expected to substantially reduce dropout rates and enhance intervention effectiveness [30].

### Limitations and Future Research

This study has several limitations. First, few of the included trials provided detailed information on software quality or reasons for dropout, which limited our ability to assess the reasons why participants stopped treatment [115]. Future studies could combine machine learning methods to predict dropout risk [116] and use participant-centered questionnaires to collect data on perceived barriers. Previous research [117-120] emphasized common reasons for dropout, including technical difficulties, lack of engagement, and perceived ineffectiveness of the intervention. Collaboration with software engineers may help optimize the digital experience and reduce technical-related attrition [121]. Additionally, methodological improvements, such as combining intention-to-treat analysis with run-in phase dropout screening [15,122], may provide more refined methods for managing early dropout.

Second, most of the digital interventions included in the studies adopted limited forms, such as videos, virtual characters, or text messages, and lacked interactive features. Incorporating gamification elements may enhance user engagement [123], especially when personalized to individual preferences [86,121]. Emerging evidence suggests that well-designed therapeutic video games can improve cognitive and mental health outcomes [121], even inducing neurobiological changes, including alterations in white matter microstructure [124-128].

Finally, many studies did not clearly report key methodological details, such as the degree of digitalization or level of human support. Although we conducted analyses including and excluding the “Not reported” category, the lack of such information led to inconsistent findings, preventing definitive conclusions regarding the impact of digitalization on dropout rates. Future studies should standardize reporting of intervention details, including digitalization and human support, to better understand active components and optimize strategies [121,129]. Another limitation is the high heterogeneity in the meta-analysis (I²>90%), which may reduce robustness. Despite sensitivity and moderator analyses, some variability remained unexplained, suggesting pooled effects may not apply equally across interventions, populations, or outcomes. Future research should adopt rigorous methodologies, including detailed reporting, preregistration, data sharing, and large-scale RCTs. Individual participant data meta-analyses can further clarify subgroup effects and sources of heterogeneity, improving generalizability [130].

### Conclusion

In summary, this meta-analysis systematically examined dropout rates and their predictive factors in digital psychosocial interventions for adult illicit drug users, aiming to provide a comprehensive picture of the research landscape in this field. The results indicate that both short-term and long-term adherence to interventions are characterized by considerable complexity. In the short term, dropout rates were primarily associated with employment status, baseline clinical diagnoses, baseline primary substance use, and intervention frequency. Over longer follow-up periods, marital status, baseline drug use frequency, and recruitment source emerged as key predictors. These findings suggest the need for further investigation into factors that contradict common assumptions or remain insufficiently reported in the literature, as well as greater standardization in the design, measurement, and reporting of randomized controlled trials to improve research quality. Moreover, more attention should be given to tailoring interventions for specific populations, particularly through the design of intervention functions and modules. Continued exploration in these areas will contribute to better supporting patients’ long-term recovery.

## Supplementary material

10.2196/77853 Multimedia Appendix 1 Search strategy.

10.2196/77853 Multimedia Appendix 2 Risk of bias.

10.2196/77853 Checklist 1 PRISMA 2020 checklist.

## Abbreviations

CMA 4.0: Comprehensive Meta-Analysis software
NR: Not reported
PRISMA: Preferred Reporting Items for Systematic Reviews and Meta-Analyses
ROB 2.0: Cochrane risk of bias tool
