# Supplementary material for: Prevalence of Dropout and Influencing Factors in Digital Psychosocial Intervention Trials for Adult Illicit Substance Users: Systematic Review and Meta-Analysis
Source: J Med Internet Res. 2025 Oct 10;27:e77853. doi: 10.2196/77853 (PMC12513713; doi:10.2196/77853)
Supplement: Multimedia Appendix 1 [file jmir-v27-e77853-s001.doc]

**Multimedia Appendix 1. Search strategy**

1. Cochrane Central Register of Controlled Trials

#1 ((digital OR email OR telephone OR phone OR cellphone* OR smartphone* OR web OR "web based" OR website* OR internet OR "internet based" OR online OR electronic OR messag* OR SMS OR text* OR (mobile NEXT health) OR (mobile NEXT technolog*) OR mhealth OR "e-mental health" OR etherapy OR "e health" OR (electronic NEXT reminder*) OR "new media" OR "Social Media" OR "electronic media" OR app OR apps OR application* OR laptop* OR pda OR (personal NEXT digital NEXT assistant*) OR (video NEXT game*))):ti,ab,kw

#2 (((Psychotherapy OR (Complementary NEXT Therap*) OR ((psychologic* OR psychodynamic OR psychosocial) AND (interven* OR treat* OR therap*)) OR ((cognit* OR behavior*) AND (treat* OR interven* OR therap* OR manag*)) OR relax* OR hypno* OR meditat* OR desensiti* OR (anxiety AND manag*) OR biofeedback OR (Patient NEXT Education) OR ((client OR patient) AND educat*) OR (cognit* AND restructur*) OR distraction OR (expressive AND disclosure) OR (group NEXT support) OR (group NEXT therap*) OR imagery OR mindfulness OR (progressive NEXT muscle NEXT relaxation) OR psychoeducation OR reframing OR (Self-Help NEXT Group*) OR ((self NEXT help) AND technique* ) OR (skill NEXT training) OR (Social NEXT Support) OR (support NEXT group*) OR (supportive NEXT therap*) OR visualization OR visualisation))):ti,ab,kw

#3 (((substance-related NEXT disorder*) OR (drug NEXT abuse) OR (drug NEXT use) OR (drug NEXT misuse) OR addicti* OR (drug NEXT dependence) OR (substance NEXT use) OR polydrug OR heroin OR cocaine OR crack OR morphine OR opium OR opiate OR opioid OR benzodiazepine OR narcotic* OR ecstasy)):ti,ab,kw

#4 (("clinical trials" or "crossover procedure" or "double blind procedure" or placebo or randomization or "random sample" or "single blind procedure" OR "randomized controlled trial")):ti,ab,kw

#5 #1 AND #2 AND #3 AND #4 in Trials

#6 English:la

#7 #5 AND #6

1. Pubmed

("cell phone"[MeSH Terms] OR "Text Messaging"[MeSH Terms] OR "Reminder Systems"[MeSH Terms] OR "internet"[MeSH Terms] OR "Social Media"[MeSH Terms] OR "Mobile Applications"[MeSH Terms] OR "computers, handheld"[MeSH Terms] OR "Software"[MeSH Terms] OR "Electronic Mail"[MeSH Terms] OR "Video Games"[MeSH Terms] OR "Social NetwORking"[MeSH Terms] OR "digital"[Title/Abstract] OR "email"[Title/Abstract] OR "e-mail"[Title/Abstract] OR "telephone"[Title/Abstract] OR "phone"[Title/Abstract] OR "cellphone*"[Title/Abstract] OR "smartphone*"[Title/Abstract] OR "web"[Title/Abstract] OR "web-based"[Title/Abstract] OR "website*"[Title/Abstract] OR "internet"[Title/Abstract] OR "internet-based"[Title/Abstract] OR "online"[Title/Abstract] OR "electronic"[Title/Abstract] OR "messag*"[Title/Abstract] OR "SMS"[Title/Abstract] OR "text*"[Title/Abstract] OR "mobile health"[Title/Abstract] OR "mobile technolog*"[Title/Abstract] OR "mhealth"[Title/Abstract] OR "e-mental health"[Title/Abstract] OR "etherapy"[Title/Abstract] OR "e-health"[Title/Abstract] OR "electronic reminder*"[Title/Abstract] OR "new media"[Title/Abstract] OR "Social Media"[Title/Abstract] OR "electronic media"[Title/Abstract] OR "app"[Title/Abstract] OR "apps"[Title/Abstract] OR "application*"[Title/Abstract] OR "laptop*"[Title/Abstract] OR "pda"[Title/Abstract] OR "personal digital assistant*"[Title/Abstract] OR "video game*"[Title/Abstract]) AND ("Psychotherapy"[Title/Abstract] OR "complementary therap*"[Title/Abstract] OR (("psychologic*"[Title/Abstract] OR "psychodynamic"[Title/Abstract] OR "psychosocial"[Title/Abstract]) AND ("interven*"[Title/Abstract] OR "treat*"[Title/Abstract] OR "therap*"[Title/Abstract])) OR (("cognit*"[Title/Abstract] OR "behavior*"[Title/Abstract]) AND ("treat*"[Title/Abstract] OR "interven*"[Title/Abstract] OR "therap*"[Title/Abstract] OR "manag*"[Title/Abstract])) OR "relax*"[Title/Abstract] OR "hypno*"[Title/Abstract] OR "meditat*"[Title/Abstract] OR "desensiti*"[Title/Abstract] OR ("anxiety"[Title/Abstract] AND "manag*"[Title/Abstract]) OR "biofeedback"[Title/Abstract] OR "Patient Education"[Title/Abstract] OR (("client"[Title/Abstract] OR "patient"[Title/Abstract]) AND "educat*"[Title/Abstract]) OR ("cognit*"[Title/Abstract] AND "restructur*"[Title/Abstract]) OR "distraction"[Title/Abstract] OR ("expressive"[Title/Abstract] AND "disclosure"[Title/Abstract]) OR "group support"[Title/Abstract] OR "group therap*"[Title/Abstract] OR "imagery"[Title/Abstract] OR ("mind s"[All Fields] OR "minded"[All Fields] OR "mindful"[All Fields] OR "mindfulness"[MeSH Terms] OR "mindfulness"[All Fields] OR "minding"[All Fields] OR "minds"[All Fields]) OR "progressive muscle relaxation"[Title/Abstract] OR "psychoeducation"[Title/Abstract] OR "reframing"[Title/Abstract] OR "self help group*"[Title/Abstract] OR ("self help"[Title/Abstract] AND "technique*"[Title/Abstract]) OR "skill training"[Title/Abstract] OR "Social Support"[Title/Abstract] OR "support group*"[Title/Abstract] OR "supportive therap*"[Title/Abstract] OR "visualization"[Title/Abstract] OR "visualisation"[Title/Abstract]) AND ("substance related disorder*"[Title/Abstract] OR "drug abuse"[Title/Abstract] OR "drug use"[Title/Abstract] OR "drug misuse"[All Fields] OR "addicti*"[Title/Abstract] OR "drug dependence"[Title/Abstract] OR "substance use"[Title/Abstract] OR "polydrug"[Title/Abstract] OR "heroin"[Title/Abstract] OR "cocaine"[Title/Abstract] OR "crack"[Title/Abstract] OR "morphine"[Title/Abstract] OR "opium"[Title/Abstract] OR "opiate"[Title/Abstract] OR "opioid"[Title/Abstract] OR "benzodiazepine"[Title/Abstract] OR "narcotic*"[Title/Abstract] OR "ecstasy"[Title/Abstract]) AND ((("clinical trials"[Title/Abstract] OR "crossover procedure"[Title/Abstract] OR "double blind procedure"[Title/Abstract] OR "placebo"[Title/Abstract] OR "randomization"[Title/Abstract] OR "random sample"[Title/Abstract] OR "single blind procedure"[Title/Abstract]) AND "randomized controlled trial"[Publication Type]) OR "randomized controlled trial"[Title/Abstract])

Filters applied: Clinical Trial, Randomized Controlled Trial, English.

1. PsycINFO

TI ( ("cell phone"OR "Text Messaging"OR "Reminder Systems"OR "internet"OR "Social Media"OR "Mobile Applications"OR "computers, handheld"OR "Software"OR "Electronic Mail"OR "Video Games"OR "Social NetwORking"OR "digital" OR "email" OR "e-mail" OR "telephone" OR "phone" OR "cellphone*" OR "smartphone*" OR "web" OR "web-based" OR "website*" OR "internet" OR "internet-based" OR "online" OR "electronic" OR "messag*" OR "SMS" OR "text*" OR "mobile health" OR "mobile technolog*" OR "mhealth" OR "e-mental health" OR "etherapy" OR "e-health" OR "electronic reminder*" OR "new media" OR "Social Media" OR "electronic media" OR "app" OR "apps" OR "application*" OR "laptop*" OR "pda" OR "personal digital assistant*" OR "video game*") AND ("Psychotherapy" OR "complementary therap*" OR (("psychologic*" OR "psychodynamic" OR "psychosocial") AND ("interven*" OR "treat*" OR "therap*")) OR (("cognit*" OR "behavior*") AND ("treat*" OR "interven*" OR "therap*" OR "manag*")) OR "relax*" OR "hypno*" OR "meditat*" OR "desensiti*" OR ("anxiety" AND "manag*") OR "biofeedback" OR "Patient Education" OR (("client" OR "patient") AND "educat*") OR ("cognit*" AND "restructur*") OR "distraction" OR ("expressive" AND "disclosure") OR "group support" OR "group therap*" OR "imagery" OR ("mind s" OR "minded" OR "mindful" OR "mindfulness"OR "mindfulness" OR "minding" OR "minds") OR "progressive muscle relaxation" OR "psychoeducation" OR "reframing" OR "self help group*" OR ("self help" AND "technique*") OR "skill training" OR "Social Support" OR "support group*" OR "supportive therap*" OR "visualization" OR "visualisation") AND ("substance related disorder*" OR "drug abuse" OR "drug use" OR "drug misuse" OR "addicti*" OR "drug dependence" OR "substance use" OR "polydrug" OR "heroin" OR "cocaine" OR "crack" OR "morphine" OR "opium" OR "opiate" OR "opioid" OR "benzodiazepine" OR "narcotic*" OR "ecstasy") AND ("clinical trials" or "crossover procedure" or "double blind procedure" or placebo or randomization or "random sample" or "single blind procedure" OR "randomized controlled trial") ) OR KW ( ("cell phone"OR "Text Messaging"OR "Reminder Systems"OR "internet"OR "Social Media"OR "Mobile Applications"OR "computers, handheld"OR "Software"OR "Electronic Mail"OR "Video Games"OR "Social NetwORking"OR "digital" OR "email" OR "e-mail" OR "telephone" OR "phone" OR "cellphone*" OR "smartphone*" OR "web" OR "web-based" OR "website*" OR "internet" OR "internet-based" OR "online" OR "electronic" OR "messag*" OR "SMS" OR "text*" OR "mobile health" OR "mobile technolog*" OR "mhealth" OR "e-mental health" OR "etherapy" OR "e-health" OR "electronic reminder*" OR "new media" OR "Social Media" OR "electronic media" OR "app" OR "apps" OR "application*" OR "laptop*" OR "pda" OR "personal digital assistant*" OR "video game*") AND ("Psychotherapy" OR "complementary therap*" OR (("psychologic*" OR "psychodynamic" OR "psychosocial") AND ("interven*" OR "treat*" OR "therap*")) OR (("cognit*" OR "behavior*") AND ("treat*" OR "interven*" OR "therap*" OR "manag*")) OR "relax*" OR "hypno*" OR "meditat*" OR "desensiti*" OR ("anxiety" AND "manag*") OR "biofeedback" OR "Patient Education" OR (("client" OR "patient") AND "educat*") OR ("cognit*" AND "restructur*") OR "distraction" OR ("expressive" AND "disclosure") OR "group support" OR "group therap*" OR "imagery" OR ("mind s" OR "minded" OR "mindful" OR "mindfulness"OR "mindfulness" OR "minding" OR "minds") OR "progressive muscle relaxation" OR "psychoeducation" OR "reframing" OR "self help group*" OR ("self help" AND "technique*") OR "skill training" OR "Social Support" OR "support group*" OR "supportive therap*" OR "visualization" OR "visualisation") AND ("substance related disorder*" OR "drug abuse" OR "drug use" OR "drug misuse" OR "addicti*" OR "drug dependence" OR "substance use" OR "polydrug" OR "heroin" OR "cocaine" OR "crack" OR "morphine" OR "opium" OR "opiate" OR "opioid" OR "benzodiazepine" OR "narcotic*" OR "ecstasy") AND ("clinical trials" or "crossover procedure" or "double blind procedure" or placebo or randomization or "random sample" or "single blind procedure" OR "randomized controlled trial") ) OR AB ( ("cell phone"OR "Text Messaging"OR "Reminder Systems"OR "internet"OR "Social Media"OR "Mobile Applications"OR "computers, handheld"OR "Software"OR "Electronic Mail"OR "Video Games"OR "Social NetwORking"OR "digital" OR "email" OR "e-mail" OR "telephone" OR "phone" OR "cellphone*" OR "smartphone*" OR "web" OR "web-based" OR "website*" OR "internet" OR "internet-based" OR "online" OR "electronic" OR "messag*" OR "SMS" OR "text*" OR "mobile health" OR "mobile technolog*" OR "mhealth" OR "e-mental health" OR "etherapy" OR "e-health" OR "electronic reminder*" OR "new media" OR "Social Media" OR "electronic media" OR "app" OR "apps" OR "application*" OR "laptop*" OR "pda" OR "personal digital assistant*" OR "video game*") AND ("Psychotherapy" OR "complementary therap*" OR (("psychologic*" OR "psychodynamic" OR "psychosocial") AND ("interven*" OR "treat*" OR "therap*")) OR (("cognit*" OR "behavior*") AND ("treat*" OR "interven*" OR "therap*" OR "manag*")) OR "relax*" OR "hypno*" OR "meditat*" OR "desensiti*" OR ("anxiety" AND "manag*") OR "biofeedback" OR "Patient Education" OR (("client" OR "patient") AND "educat*") OR ("cognit*" AND "restructur*") OR "distraction" OR ("expressive" AND "disclosure") OR "group support" OR "group therap*" OR "imagery" OR ("mind s" OR "minded" OR "mindful" OR "mindfulness"OR "mindfulness" OR "minding" OR "minds") OR "progressive muscle relaxation" OR "psychoeducation" OR "reframing" OR "self help group*" OR ("self help" AND "technique*") OR "skill training" OR "Social Support" OR "support group*" OR "supportive therap*" OR "visualization" OR "visualisation") AND ("substance related disorder*" OR "drug abuse" OR "drug use" OR "drug misuse" OR "addicti*" OR "drug dependence" OR "substance use" OR "polydrug" OR "heroin" OR "cocaine" OR "crack" OR "morphine" OR "opium" OR "opiate" OR "opioid" OR "benzodiazepine" OR "narcotic*" OR "ecstasy") AND ("clinical trials" or "crossover procedure" or "double blind procedure" or placebo or randomization or "random sample" or "single blind procedure" OR "randomized controlled trial") )

**Limiters** - Limit to Peer Reviewed; English

**Search modes** - Boolean/Phrase

1. Embase

(( "digital" OR "email" OR "e-mail" OR "telephone" OR "phone" OR "cellphone*" OR "smartphone*" OR "web" OR "web-based" OR "website*" OR "internet" OR "internet-based" OR "online" OR "electronic" OR "messag*" OR "SMS" OR "text*" OR "mobile health" OR "mobile technolog*" OR "mhealth" OR "e-mental health" OR "etherapy" OR "e-health" OR "electronic reminder*" OR "new media" OR "Social Media" OR "electronic media" OR "app" OR "apps" OR "application*" OR "laptop*" OR "pda" OR "personal digital assistant*" OR "video game*" )):ti,ab,kw AND ((( Psychotherapy OR "Complementary Therap*" OR ( ( psychologic* OR psychodynamic OR psychosocial ) AND ( interven* OR treat* OR therap* ) ) OR ( ( cognit* OR behavior* ) AND ( treat* OR interven* OR therap* OR manag* ) ) OR relax* OR hypno* OR meditat* OR desensiti* OR ( anxiety AND manag* ) OR biofeedback OR "Patient Education" OR ( ( client OR patient ) AND educat* ) OR ( cognit* AND restructur* ) OR distraction OR ( expressive AND disclosure ) OR "group support" OR "group therap*" OR imagery OR 'mindfulness OR progressive muscle relaxation' OR psychoeducation OR reframing OR "Self-Help Group*" OR ( "self help" AND technique* ) OR "skill training" OR "Social Support" OR "support group*" OR "supportive therap*" OR visualization OR visualisation )):ti,ab,kw) AND ((( "substance-related disorder*" OR "drug abuse" OR "drug use" OR "drug misuse" OR "addicti*" OR "drug dependence" OR "substance use" OR polydrug OR heroin OR cocaine OR crack OR morphine OR opium OR opiate OR opioid OR benzodiazepine OR narcotic* OR ecstasy )):ti,ab,kw) AND ((English):la) AND [Randomized Controlled Trial]/lim

1. Web of science

(TS=( "digital" OR "email" OR "e-mail" OR "telephone" OR "phone" OR "cellphone*" OR "smartphone*" OR "web" OR "web-based" OR "website*" OR "internet" OR "internet-based" OR "online" OR "electronic" OR "messag*" OR "SMS" OR "text*" OR "mobile health" OR "mobile technolog*" OR "mhealth" OR "e-mental health" OR "eutherapy" OR "e-health" OR "electronic reminder*" OR "new media" OR "Social Media" OR "electronic media" OR "app" OR "apps" OR "application*" OR "laptop*" OR "pda" OR "personal digital assistant*" OR "video game*" )) and (TS=(( Psychotherapy OR "Complementary Therap*" OR ( ( psychologic* OR psychodynamic OR psychosocial ) AND ( interven* OR treat* OR therap* ) ) OR ( ( cognit* OR behavior* ) AND ( treat* OR interven* OR therap* OR manag* ) ) OR relax* OR hypno* OR meditat* OR desensiti* OR ( anxiety AND manag* ) OR biofeedback OR "Patient Education" OR ( ( client OR patient ) AND educat* ) OR ( cognit* AND restructur* ) OR distraction OR ( expressive AND disclosure ) OR "group support" OR "group therap*" OR imagery OR 'mindfulness OR progressive muscle relaxation' OR psychoeducation OR reframing OR "Self-Help Group*" OR ( "self help" AND technique* ) OR "skill training" OR "Social Support" OR "support group*" OR "supportive therap*" OR visualization OR visualisation ))) and (TS=( "substance-related disorder*" OR "drug abuse" OR "drug use" OR "drug misuse" OR "addicti*" OR "drug dependence" OR "substance use" OR polydrug OR heroin OR cocaine OR crack OR morphine OR opium OR opiate OR opioid OR benzodiazepine OR narcotic* OR ecstasy )) and (TS=("clinical trials" or "crossover procedure" or "double blind procedure" or placebo or randomization or "random sample" or "single blind procedure" OR "randomized controlled trial")) not (TI=("systematic review" or meta-analysis))

**Refined By: Languages: English**. **NOT Document Types: Review Article**
