# Supplementary figures and images for: Prevalence of Dropout and Influencing Factors in Digital Psychosocial Intervention Trials for Adult Illicit Substance Users: Systematic Review and Meta-Analysis
Source: J Med Internet Res. 2025 Oct 10;27:e77853. doi: 10.2196/77853 (PMC12513713; doi:10.2196/77853)

**Multimedia Appendix 2. Risk of bias**


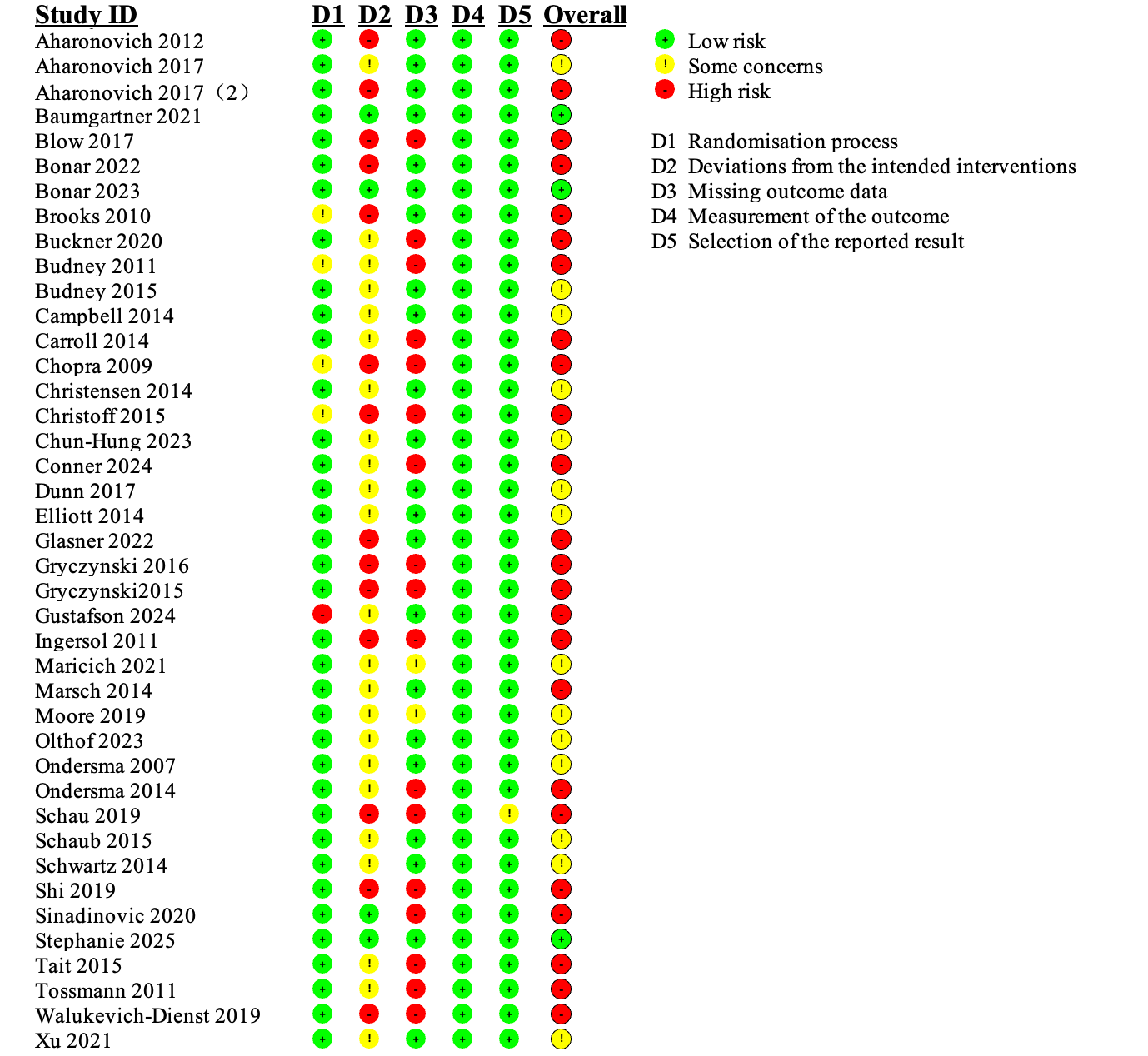


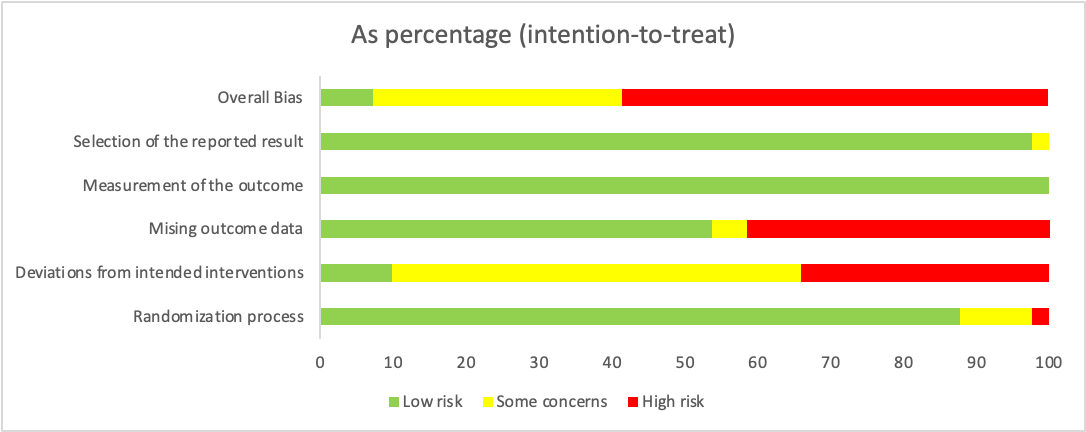

Supplement: Multimedia Appendix 2 [file jmir-v27-e77853-s002.docx]
